# Risk of intracranial haemorrhage in patients with acute ischaemic stroke and prior antiplatelet therapy

**DOI:** 10.1093/esj/23969873251369755

**Published:** 2026-01-01

**Authors:** Lukas Nussbaum, Sabine Schaedelin, Leo Bonati, Marcel Arnold, David J Seiffge, Martina Goeldlin, Stefan Engelter, Alexandros A Polymeris, Timo Kahles, Krassen Nedeltchev, Georg Kägi, Davide Strambo, Alexander Salerno, Emmanuel Carrera, Susanne Wegener, Carlo Cereda, Manuel Bolognese, Lehel-Barna Lakatos, Andrea Humm, Friedrich Medlin, Nils Peters, Dennis Thumm, Sylvan Albert, Maria Ligon, Christian Berger, Marie-Luise Mono, Susanne Renaud, Julien Niederhauser, Alexander Tarnutzer, Nicole Bruni, Mira Katan, Gian Marco De Marchis, Philippe Lyrer

**Affiliations:** Department of Neurology and Stroke Centre, University Hospital Basel and University of Basel, Basel, Switzerland; Department of Clinical Research, Clinical Trial Unit, University Hospital Basel, Basel, Switzerland; Reha Rheinfelden, Rheinfelden and University of Basel, Basel, Switzerland; Department of Neurology, Inselspital University Hospital Bern and University of Bern, Bern, Switzerland; Department of Neurology, Inselspital University Hospital Bern and University of Bern, Bern, Switzerland; Department of Neurology, Inselspital University Hospital Bern and University of Bern, Bern, Switzerland; Department of Geriatric Medicine Felix Platter, University Hospital Basel and University of Basel, Basel, Switzerland; Department of Neurology and Stroke Centre, University Hospital Basel and University of Basel, Basel, Switzerland; Department of Neurology and Stroke Centre, Cantonal Hospital Aarau, Aarau and University of Basel, Basel, Switzerland; Department of Neurology and Stroke Centre, Cantonal Hospital Aarau, Aarau and University of Bern, Bern, Switzerland; Department of Neurology and Stroke Centre, Cantonal Hospital St Gallen, Sankt Gallen, Switzerland; Department of Neurology, Lausanne University Hospital and University of Lausanne, Lausanne, Switzerland; Department of Neurology, Lausanne University Hospital and University of Lausanne, Lausanne, Switzerland; Department of Neurology, Hôpitaux Universitaires Genève, Geneve, Switzerland; Department of Neurology, University Hospital Zurich and University of Zurich, Zurich, Switzerland; Stroke Center EOC, Ospedale Regionale di Lugano, Lugano, Ticino, Switzerland; Department of Neurology, Luzerner Kantonsspital, Luzern, Switzerland; Department of Neurology, Luzerner Kantonsspital, Luzern, Switzerland; Stroke Unit and Division of Neurology, HFR Fribourg Cantonal Hospital, Fribourg, Switzerland; Stroke Unit and Division of Neurology, HFR Fribourg Cantonal Hospital, Fribourg, Switzerland; Stroke Center, Klinik Hirslanden, Stroke Center, Zurich and University of Basel, Basel, Switzerland; Stroke Center, Klinik Hirslanden, Stroke Center, Zurich and University of Basel, Basel, Switzerland; Department of Neurology, Kantonsspital Chur, Chur, Switzerland; Department of Neurology, Kantonsspital Chur, Chur, Switzerland; Stroke Unit, Cantonal Hospital Grabs, Grabs Switzerland; Department of Neurology, Stadtspital Zurich Triemli, Zurich, Switzerland; Stroke Unit and Division of Neurology, Network Hospital Neuchâtel, Neuchatel, Switzerland; Stroke Unit, Hopital de Nyon, Nyon, Switzerland; Department of Neurology, Kantonsspital Baden, Baden and University of Zurich, Zurich, Switzerland; Department of Clinical Research, Clinical Trial Unit, University Hospital Basel, Basel, Switzerland; Department of Neurology and Stroke Centre, University Hospital Basel and University of Basel, Basel, Switzerland; Department of Neurology and Stroke Center, Cantonal Hospital St Gallen, St. Gallen and University of Basel, Basel, Switzerland; Department of Neurology and Stroke Centre, University Hospital Basel and University of Basel, Basel, Switzerland

**Keywords:** Ischaemic stroke, platelet aggregation inhibitors, intracranial haemorrhages, aspirin, clopidogrel

## Abstract

**Introduction:**

Whether patients with acute ischaemic stroke (AIS) and prior antiplatelet therapy (APT) are at higher risk of symptomatic intracranial haemorrhage (sICH) has not been established. This study aimed to determine whether prior APT increases the risk of bleeding.

**Methods:**

41,113 patients treated for AIS in Switzerland between 2014 and 2022 were analysed. The primary outcome was sICH, and secondary outcomes were recurrent ischaemic stroke (IS), all-cause mortality, functional outcome at discharge and 90 days. Patients grouped by prior APT: no APT (nAPT), single APT (SAPT), and dual APT (DAPT) were analysed using logistic and Cox proportional hazards regression. Confounding variables, including revascularisation treatment, were accounted for using multivariable adjustment and matching, and a time-to-event analysis was performed.

**Results:**

In the adjusted analysis at 90 days, patients with nAPT versus SAPT had a decreased risk of sICH (aOR 0.75 (0.62–0.90), *p* < 0.01), while these occurred within the first days. There was no difference in the risk of recurrent IS, all-cause mortality, or functional outcome. Patients with DAPT had no higher risk of sICH or mortality than those with SAPT, but a higher occurrence of recurrent IS (aOR 1.41 (1.12–1.77), *p* < 0.01) and worse functional outcome (aOR 1.18 (1.07–1.31), *p* < 0.01). The results were consistent after adjusting for revascularisation treatment.

**Conclusions:**

Patients with nAPT had a lower risk of sICH than those with SAPT. Patients with DAPT have a higher risk of recurrent IS and worse functional outcome respectively. The majority of sICH occurred within the first days after admission.

## Graphical Abstract

**Graphical Abstract:**
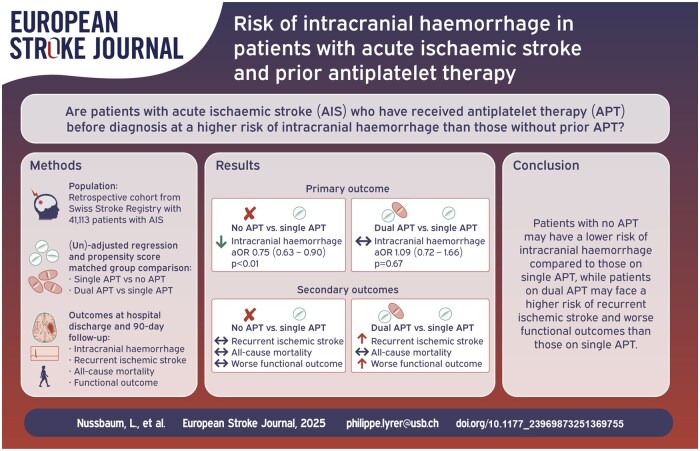

## Introduction

Acute ischaemic stroke (AIS) remains a leading cause of disability and mortality worldwide, particularly among the elderly. Antiplatelet therapy (APT) is commonly used to reduce the risk of AIS in high-risk patients, including those with a history of AIS or transient ischaemic attack (TIA), and for various cardiological indications. While APT is effective in reducing the risk of thromboembolic events, it may also increase the risk of symptomatic intracranial haemorrhage (sICH), with implications for patient outcomes.

Several studies addressed the relationship between APT and sICH in selected patients with AIS who underwent reperfusion treatments, such as intravenous thrombolysis (IVT) or endovascular thrombectomy (EVT). Heterogeneous results have been reported, with some studies suggesting that prior APT increases the risk of sICH in patients with AIS,^1–6^ while others have found no significant association.^7–11^ In patients treated with intravenous thrombolysis (IVT), systematic reviews and meta-analyses have shown that prior APT increases the risk of sICH only in unadjusted analyses. The association was no longer significant once the analyses were adjusted for various confounders.^12–14^ Despite these ambiguous results, most studies have focussed on patients undergoing reperfusion treatments, and data on the broader population of patients with ischaemic stroke are lacking.

Indeed, many patients with AIS do not receive reperfusion treatment but are treated with antithrombotic therapies, at least such as single antiplatelet therapy (SAPT) or dual antiplatelet therapy (DAPT). The use of DAPT, particularly in patients with moderate-to-severe AIS, has become increasingly common in clinical practice.^15^ Studies such as CHANCE,^16^ POINT,^17^ and INSPIRES^18^ have examined DAPT after the index event in specific populations. However, the broader question of whether prior APT increases the risk of sICH in the population of patients with ischaemic stroke, regardless of reperfusion treatments, remains insufficiently addressed.

This study focuses on these gaps by investigating a broad cohort of patients with AIS. Specifically, we aimed to (i) determine whether patients with AIS who received SAPT or DAPT prior to diagnosis are at a higher risk for sICH than those with no prior APT and (ii) examine the association of prior APT with recurrent IS, all-cause mortality, and functional outcome.

## Patients and methods

### Study design and patient selection

All consecutive adult patients with cerebrovascular events who were treated in one of the 14 certified Stroke Units or 10 Stroke Centres in Switzerland (list of centres in Supplemental Table S1) were enrolled in the Swiss Stroke Registry (SSR), and their data were recorded in its web-based registry. The SSR was initiated to prospectively collect data for quality control in acute stroke management, which also allows addressing clinically important research questions, as done previously.^19–22^ The SSR covers approximately 85% of all stroke patients in Switzerland and is considered to be representative. Predefined variables were collected from all participating centres. Data during hospitalisation were obtained from the patient files, and the 90-day data were collected either clinically in a neurovascular consultation or through structured telephone interviews with the patient or next of kin.

For this study, data from all patients with AIS as a qualifying event between 01/2014 and 11/2022 were included. Patients who objected to the use of their data for scientific purposes, were under oral anticoagulants before the qualifying event, had missing data on medical treatment before stroke onset, or had incomplete follow-up were excluded.

Our analysis followed the STROBE guidelines for observational studies.^23^

### Exposures

The exposure was prior APT, defined as the use of aspirin, clopidogrel, ticagrelor, prasugrel, or dipyridamole. Either alone (SAPT) or in combination (DAPT).

### Outcomes

The primary outcome was symptomatic ICH, defined as radiologically visible bleeding in combination with *a* ⩾4 points worsening in the National Institutes of Health Stroke Scale (NIHSS) score, measured at hospital discharge and within 90 days after the initial event.^24^

The secondary outcomes were (1) recurrent IS, (2) all-cause mortality, and (3) functional outcome assessed using the mRS. Outcomes (1) and (2) were measured at hospital discharge and within 90 days after the initial event, and outcome (3) was measured 90 days after the initial event.

The time of occurrence of sICH, recurrent IS, and all-cause mortality was also recorded and analysed.

### Statistical analysis

Demographic variables and patient characteristics were presented as frequencies and percentages, and continuous data as median and interquartile range (IQR). Baseline data were also analysed for temporal trends in prior APT and revascularisation treatments. Baseline data of the excluded patients were presented and compared with those of the included patients.

#### Main analysis

The primary outcome (sICH) and binary secondary outcomes (recurrent IS, all-cause mortality) were analysed using both (a) logistic regression and (b) Cox regression, while the 90-day mRS was analysed using ordinal logistic regression.

To mitigate the risk of bias, a directed acyclic graph (DAG) was created using DAGitty.^25^ The details are provided in the Supplemental.

The following analyses were performed accordingly:

i. unadjusted with comparisons of nAPT versus SAPT and DAPT versus SAPT.
ii. regression analysis with covariate adjustment of the variables age, sex, mRS before the event, NIHSS at admission, prior TIA or amaurosis fugax, prior AIS or retinal infarction, prior ICH, hypertension, diabetes, hyperlipidaemia, smoking, atrial fibrillation/flutter, coronary heart disease, and documented left ventricular ejection fraction <35%, as defined in Fluri et al.^26^
iii. propensity score matching using the R package MatchIt.^27^

Details are provided in the Supplemental.

#### Sensitivity analysis

As a sensitivity analysis, time to sICH, recurrent IS, and all-cause mortality were analysed as time-to-event analyses using a Cox proportional hazard model. The proportional hazards assumption was checked by visually inspecting the Schoenfeld’s residuals. The results of the time-to-event analyses were compared with those of the main analysis. The details are provided in the Supplemental.

#### Analysis of possible mediation effect of revascularization treatments

To further assess whether the estimated effect of prior ATP was mediated by acute treatment after the qualifying event, revascularization treatment was analysed for a possible mediator effect, as described by Baron and Kenny.^28,29^ Details are given in the Supplemental. IVT was defined as the use of intravenous recombinant tissue plasminogen activator. EVT was defined as the use of any mechanical intra-arterial treatment with or without intra-arterial recombinant tissue plasminogen activator or intra-arterial urokinase.

#### Handling of missing values

The missing values were summarised for each variable. Imputation was performed using Chained Equations in the R package MICE.^30^ If patients died within the first 3 months, we did not impute outcomes sICH and stroke. Details are given in the Supplemental.

The results are presented as estimated effect sizes with measures of variance and precision. The hypotheses were predefined in the statistical analysis plan. The adjusting variables were predefined to minimise bias and allow causal interpretation, given the assumptions of positivity, data quality, and DAG appropriateness.^31^ However, as the data used in this study are part of a cohort which has been analysed by the same study team before regarding different study questions, we consider these results as post-hoc analyses. *p*-Values should be interpreted as continuous measures informing new hypotheses, alongside estimated effect sizes.^32^

All statistical analyses were performed with R version 4.1.2.^33^

#### Ethical approval

Institutional Review Board approval was obtained from the Ethikkommission Nordwest- und Zentralschweiz, project-ID 2022-02045. All procedures performed in this study complied with the 1964 Declaration of Helsinki and subsequent amendments or comparable ethical standards.

## Results

### Patient characteristics and revascularisation treatment

**Figure 1.**
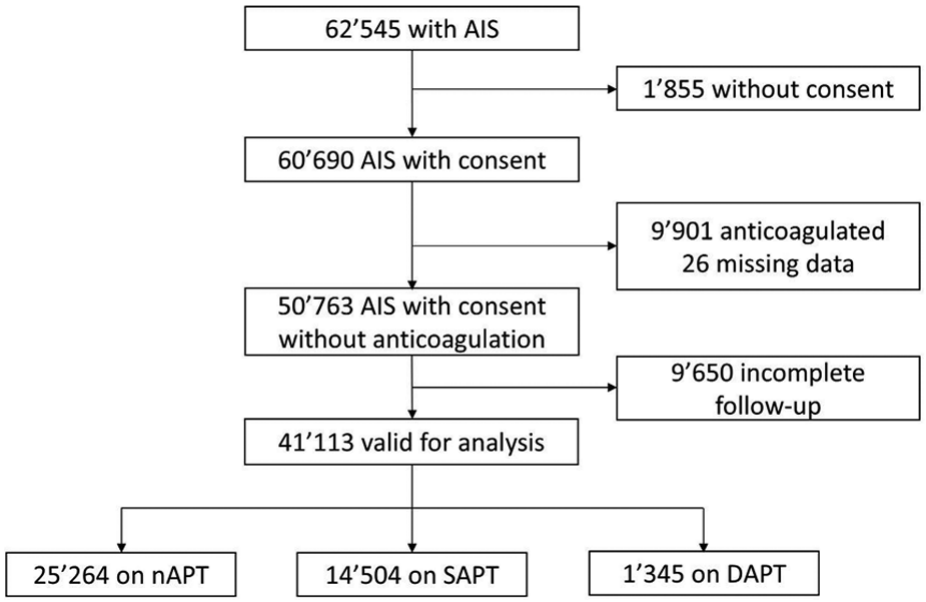
Study flowchart of patient selection from the SSR.

Out of 62,545 patients with AIS included in the SSR during the study period, 41,113 (65.7%) were eligible for analysis, while 21,432 (34.3%) were excluded for the following reasons: 1855 (3.0%) refused consent to use their data for research, 9901 (15.8%) were anticoagulated, 26 (0.1%) had missing data, and 9650 (15.4%) had incomplete follow-up (flowchart displayed in Figure 1).

The sensitivity analysis of excluded patients showed no differences in the baseline data or revascularisation treatment (Supplemental Table S2).

From the study cohort 25,264 (61.5%) had nAPT, 14,504 (35.3%) had SAPT, and 1345 (3.3%) had DAPT at AIS onset. During the observation period, the percentage of patients on DAPT did not change, whereas the percentage of patients on SAPT decreased slightly (Supplemental Table S3 and Figure S1). Patients with any APT were older, more likely to have a history of stroke or TIA, had a higher prestroke mRS score, and were more likely to have hypertension, diabetes, hyperlipidaemia, and coronary heart disease (Table 1).

**Table 1.** Patient characteristics in the three groups of interest: nAPT, SAPT, and DAPT.

	nAPT	SAPT	DAPT	SMD	Missing %
Number	25,264	14,504	1345		
Age (median, IQR)	70.8 (59.2, 80.3)	78.1 (70.3, 84.7)	74.9 (67.2, 81.7)	0.396	7.8
Sex				0.160	0.1
Male (%)	14,149 (55.9)	8768 (60.4)	908 (67.6)		
Female (%)	11,115 (44.1)	5736 (39.6)	436 (32.4)		
NIHSS at admission (median, IQR)	3.0 (1.0, 8.0)	3.0 (1.0, 8.0)	3.0 (1.0, 7.0)	0.034	0.9
mRS before event (median, IQR)	0.0 (0.0, 1.0)	0.0 (0.0, 1.0)	0.0 (0.0, 2.0)	0.239	10.6
Past cerebrovascular events					
TIA or amaurosis fugax (%)	565 (2.3)	1473 (10.2)	151 (11.3)	0.245	1.1
AIS or retinal infarction (%)	1595 (6.4)	4366 (30.3)	522 (39.1)	0.561	1.1
sICH (%)	413 (1.7)	279 (1.9)	31 (2.3)	0.032	1.1
Frequency of prior APT by substance					
Aspirin	0 (0)	12,486 (86.2)	1342 (99.8)	11.330	0.6
Clopidogrel	0 (0)	1984 (13.7)	1128 (83.9)	1.923	0.6
Prasugrel	0 (0)	12 (0.1)	46 (3.4)	0.188	0.6
Ticagrelor	0 (0)	8 (0.1)	157 (11.7)	0.353	0.7
Dipyridamole	0 (0)	6 (0)	17 (1.3)	0.114	0.7
Cerebrovascular risk factors					
Hypertension (%)	15,945 (63.9)	12,234 (84.8)	1151 (86.3)	0.356	1.7
Diabetes (%)	3859 (15.5)	4237 (29.4)	476 (35.7)	0.316	1.7
Hyperlipidaemia (%)	15,367 (61.7)	10,614 (73.7)	1049 (78.6)	0.250	1.8
Smoking (%)	5673 (22.9)	2932 (20.4)	355 (26.8)	0.100	2.2
Atrial fibrillation/flutter (%)	3704 (14.8)	2656 (18.4)	171 (12.8)	0.103	1.5
Coronary heart disease (%)	1252 (5.0)	4580 (31.8)	707 (53.0)	0.807	1.8
Low ejection fraction (%)	300 (1.7)	298 (2.9)	51 (5.4)	0.136	30.1
NIHSS at admission (median, IQR)	3.0 (1.0, 8.0)	3.0 (1.0, 8.0)	3.0 (1.0, 7.0)	0.070	0.9
0 points (%)	3964 (15.9)	1989 (13.8)	212 (15.9)		
1 point (%)	3479 (13.9)	1878 (13.0)	176 (13.2)		
2–4 points (%)	7669 (30.7)	4539 (31.5)	426 (31.9)		
5–15 points (%)	6987 (27.9)	4366 (30.3)	384 (28.8)		
16–20 points (%)	1598 (6.4)	929 (6.4)	82 (6.1)		
21–42 points (%)	1304 (5.2)	708 (4.9)	55 (4.1)		
Revascularisation treatment					
IVT (%)	7179 (28.7)	3981 (27.5)	249 (18.6)	0.160	0.6
EVT (%)	4217 (16.8)	2095 (14.5)	165 (12.3)	0.086	0.5
Secondary prevention					
Antiplatelet (%)	14,293 (57.1)	7974 (55.1)	770 (57.3)	0.03	0.6
Anticoagulant (%)	1180 (4.7)	663 (4.6)	62 (4.6)	0.004	0.7
Carotid angioplasty and stenting (%)	350 (1.4)	285 (2.0)	61 (4.7)	0.128	3.5
Other endo-revascularisation (%)	218 (1.0)	117 (0.9)	30 (2.6)	0.084	13.5
Other surgical-revascularisation (%)	68 (0.3)	52 (0.4)	8 (0.7)	0.036	13.8
Patent foramen ovale closure (%)	470 (1.9)	49 (0.3)	3 (0.2)	0.112	3.8
Outcome in hospital					
sICH in hospital (%)	332 (1.3)	244 (1.7)	21 (1.6)	0.020	0.0
Recurrent IS in hospital (%)	418 (1.7)	260 (1.8)	41 (3.0)	0.061	0.0
All-cause mortality in hospital (%)	1264 (5.0)	978 (6.7)	91 (6.8)	0.050	0.0
Hospitalisation duration (days, IQR)	6.0 (3.0, 9.0)	6.0 (4.0, 10.0)	7.0 (4.0, 12.0)	0.058	6.0
Outcome at 90-day follow-up					
sICH at 90-day follow-up (%)	388 (1.5)	278 (1.9)	26 (1.9)	0.020	0.0
Recurrent IS at 90-day follow-up (%)	892 (3.5)	681 (4.7)	96 (7.1)	0.108	0.0
All-cause mortality at 90-day follow-up (%)	2496 (9.9)	2010 (13.9)	177 (13.2)	0.082	0.0
mRS at 90-day follow-up				0.219	3.1
0 points (%)	7878 (32.3)	3545 (25.1)	283 (21.8)		
1 point (%)	5991 (24.5)	3029 (21.4)	282 (21.7)		
2 points (%)	3683 (15.1)	2122 (15.0)	245 (18.8)		
3 points (%)	2423 (9.9)	1873 (13.3)	179 (13.8)		
4 points (%)	1600 (6.6)	1288 (9.1)	118 (9.1)		
5 points (%)	314 (1.3)	258 (1.8)	15 (1.2)		
6 points (%)	2520 (10.3)	2018 (14.3)	179 (13.8)		

The rates of revascularisation treatment were similar in the three groups, except for the rate of IVT, which was lower in the DAPT group than in the SAPT group (Supplemental Figure S2). During the observation period, no time trend in the percentage of IVT and EVT was detected (Supplemental Table S3 and Figures S3 and S4). The review of IVT and EVT as possible mediators showed that not all conditions for a mediator, according to Baron and Kenny, were met.^28^ Thus, a possible association between prior APT and sICH cannot be explained primarily by revascularisation treatment.

The follow-up examinations were conducted in 49.4% during a clinical follow-up visit, in 33.4% by phone call, and in 17.2% by written discharge letter.

Using the DAG, the relationships between the individual variables and confounders were identified. This allowed the statistical methods to be correctly selected for calculating the individual outcomes. The DAG is shown in Supplemental Figure S5.

### Primary outcome

The frequency of sICH in hospital was 332 (1.3%) in patients with nAPT, 244 (1.7%) in patients with SAPT, and 21 (1.6%) in patients with DAPT. At the 90-day follow-up, the rate was 388 (1.5%) in patients with nAPT, 278 (1.9%) in patients with SAPT, and 26 (1.9%) in patients with DAPT.

The main findings were as follows: patients with nAPT appeared to have a lower risk of sICH than those with SAPT. This was observed in the hospitalisation phase (aOR 0.71 (0.59–0.87), *p* < 0.01) and at the 90-day follow-up (aOR 0.75 (0.63–0.90), *p* < 0.01). A decrease in bleeding risk was observed after adjusting for revascularisation treatment. When comparing the DAPT and SAPT groups, no increase in the risk of sICH was observed (hospitalisation aOR 1.04 (0.66–1.65), *p* = 0.86 and 90-day follow-up aOR 1.09 (0.72–1.66), *p* = 0.67). Even after adjusting for IVT and EVT, no association was observed between DAPT and sICH.

In the unadjusted analyses, the same trends were observed as in the adjusted analysis, with slightly higher ORs. Propensity score matching data showed comparable results. The time-to-event analysis showed results consistent with the main analysis.

**Figure 2.**
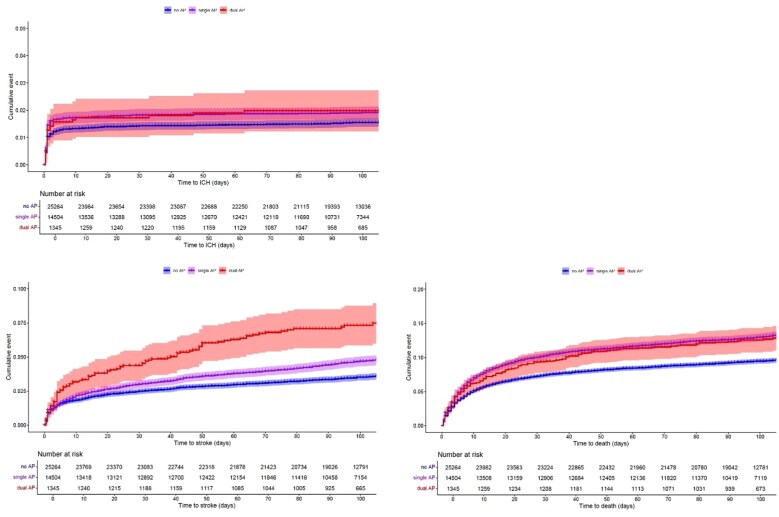
Kaplan–Meier curves showing time to sICH, recurrent ischaemic stroke, and death over a 90-day follow-up period.

The detailed results are presented in Table 2 and the forest plots in Supplemental Figure S6. The Kaplan-Meier curves are shown in Figure 2, and the full data are presented in Supplemental Table S4.

**Table 2.** Analysis of primary outcome sICH.

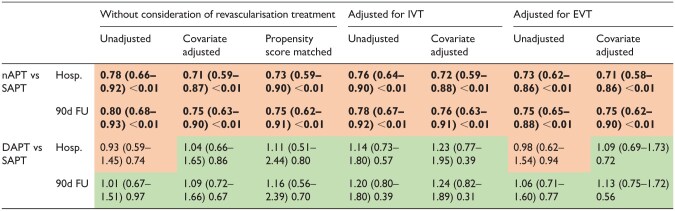

Values are OR (boxes in red OR < 1, boxes in green OR > 1), confidence intervals and *p* (bold *p* < 0.01).

### Secondary outcomes

*Recurrent ischaemic stroke*: 418 (1.7%) patients with nAPT had a recurrent IS in hospital, 260 (1.8%) patients with SAPT, and 41 (3.0%) patients with DAPT. At the 90-day follow-up, the rates were 892 (3.5%) in patients with nAPT, 681 (4.7%) in patients with SAPT, and 96 (7.1%) in patients with DAPT.

The main findings were as follows: the adjusted odds of recurrent IS in patients with nAPT compared to those with SAPT did not differ during the hospitalisation phase and 90-day follow-up. The same applied after adjusting for revascularisation treatment. In the unadjusted analysis, all 90-day outcomes showed a risk reduction. When comparing the groups treated with DAPT to those treated with SAPT, the risk of recurrent IS was higher in patients treated with DAPT (hospitalisation aOR 1.57 (1.11–2.21), *p* < 0.01 and 90-day follow-up aOR 1.41 (1.12–1.77), *p* < 0.01). This result was similar after additional adjustment for revascularisation treatment and was always higher in the hospitalisation phase than in the 90-day follow-up.

The time-to-event analysis showed consistent results with the main analysis.

*All-cause mortality*: 1264 (5.0%) patients with nAPT died in the hospital, 978 (6.7%) patients with SAPT, and 91 (6.8%) patients with DAPT. At the 90-day follow-up, the rate was 2496 (9.9%) in patients with nAPT, 2010 (13.9%) in patients with SAPT, and 177 (13.2%) in patients with DAPT.

The main findings were as follows: patients with nAPT compared to those with SAPT appeared to have a slightly lower likelihood of dying during the hospitalisation phase (aOR 0.85 (0.76–0.95), *p* < 0.01). At the 90-day follow-up, there were no relevant differences in the adjusted analysis, but in the unadjusted (OR 0.68 (0.64–0.73), *p* < 0.01). The same trend was observed for the values corrected for revascularisation treatment. When comparing the groups treated with DAPT to those treated with SAPT, there was no association observed between APT and all-cause mortality. Propensity score matching data and time-to-event analysis showed comparable results.

*Functional Outcome after 90 days*: In terms of functional outcome measured by mRS at 90 days, a favourable neurological outcome (mRS 0–2) was seen in 17,552 (72%) patients with nAPT, 8696 (62%) patients with SAPT, and 810 (62%) patients with DAPT. 4337 (18%) patients with nAPT, 3419 (24%) patients with SAPT, and 312 (24%) patients with DAPT had an unfavourable neurological outcome (mRS 3–5).

The main findings were as follows: In patients with nAPT compared with those with SAPT, there was no relevant difference in functional outcomes after 90 days, even after adjusting for revascularisation treatment. Only the unadjusted analysis showed poorer functional outcomes. Patients with DAPT compared to those with SAPT were slightly more likely to score one point higher on the mRS (aOR 1.18 (1.07–1.31), *p* < 0.01) even after adjusting for revascularisation treatment. Propensity score matching data showed comparable results.

All secondary outcome results are presented in Table 3 and the forest plots in Supplemental Figure S7. The Kaplan-Meier curves are shown in Figure 2, and the full data are presented in Supplemental Table S5.

**Table 3.** Analysis of secondary outcomes.

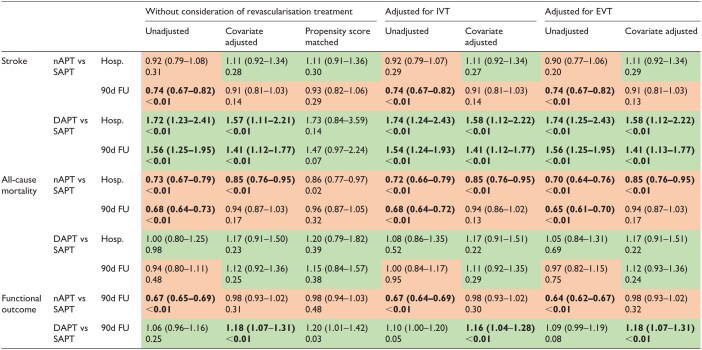

Values are OR (boxes in red OR < 1, boxes in green OR > 1), confidence intervals and *p* (bold *p* < 0.01).

## Discussion

The data suggest that patients with prior nAPT may have a lower risk of sICH than those with SAPT, without a difference in the risk of recurrent IS. This was observed both during hospitalisation and at the 90-day follow-up, regardless of revascularisation treatment. Conversely, this means that patients with prior SAPT have an increased risk of sICH compared to those with nAPT. The majority of sICH occurred within the few first days of acute hospitalisation. As mentioned in the introduction, previous studies mostly included only patients who underwent IVT or EVT under prior APT but no patients who received other treatments. In the case of prior APT, they only made a difference between any APT and nAPT.

In studies with IVT patients, the outcome sICH was partially increased in patients with prior APT compared to patients without APT: Pan et al.^34^ aOR 1.65 (1.44–1.90), Luo et al.^35^ aOR 1.21 (1.02–1.44), Naeem et al.^36^ aOR 1.78 (1.48–2.13). Others did not show a higher risk: Tsivgoulis et al.^13^ reported an aOR of 1.67 (0.75–3.72), and Malhotra et al.^10^ reported an aOR of 2.03 (0.75–5.52). In patients who underwent EVT, a new meta-analysis by Wu et al.^37^ found an increased bleeding risk, with a reported aOR of 1.31 (0.95–1.81).

While there was a higher risk of sICH, there was no increased risk of recurrent IS in the secondary outcomes (aOR 0.91 (0.81–1.03)).

Previous SAPT was associated with increased overall mortality during hospitalisation. No differences were observed at the 90-day follow-up. Therefore, it appears that these patients do not die more often but earlier (during the hospitalisation phase, which was minimally longer in patients with SAPT than in those with nAPT).

Further, the results indicate that patients who received prior DAPT may have a higher risk of recurrent IS without an increased risk of sICH during hospitalisation and at the 90-day follow-up, regardless of revascularisation treatment. Only a few studies have examined the risk of recurrent IS in patients with prior APT. A smaller study from 2021 with 2066 patients found no increased risk of recurrent IS after adjustment.^38^ Recently, a paper from the same SSR was published with a focus on ischaemic stroke despite APT without distinguishing between SAPT and DAPT use. Antiplatelet use was associated with a higher risk of recurrent stroke at 3 months, but it was not directly linked to adverse functional outcomes or all-cause mortality.^39^

Patients with prior DAPT experience more recurrent ischaemic strokes but not more sICH than patients with prior SAPT, which could be a reason for reconsidering the widespread hesitance to administer IVT or EVT in patients on DAPT. Further research should focus on the overall risk-benefit profile for IVT or EVT in patients on DAPT.

General thoughts: The differences in baseline data were consistent with other prospective registry studies of APT and stroke, such as the study by Sylaja et al.,^38^ and the matched data were well balanced. The differences in stroke, TIA, and coronary heart disease could be explained by the association between the first event or myocardial infarction and APT prescription.

Patients on DAPT were less likely to receive IVT or EVT than those on SAPT (corrected for covariates). It is unclear whether the treating physicians were more reluctant to prescribe IVT or EVT in patients on DAPT. Other factors favouring the decision not to provide IVT or EVT may have been present, such as additional comorbidities, such as recent myocardial infarctions that are treated with DAPT. This could have hidden consequences for patients on DAPT, although the European Stroke Organisation international guidelines from 2021 recommend IVT in both SAPT and DAPT.^40^

### Strengths and weaknesses

The strengths of this analysis were that the data in the SSR did not contain a selection bias for inclusion in the registry and that the data were collected prospectively for quality control. This resulted in a large dataset from different centres over several years, with high validity. The analysis was performed for three patient groups (DAPT, SAPT, and nAPT) and adjusted for revascularisation treatment.

As the dataset was collected prospectively in a predefined manner, the study team did not influence the selection of the collected parameters, which could be a limitation of the study. The variables were defined at the beginning of the 2010s decade. All new findings and questions gained since then and the resulting variables of interest were not included in the database. This includes the duration of previous medication intake and adherence to treatment. While the pharmacological effects of platelet aggregation inhibitors suggest a rapid onset of action, the lack of detailed information on treatment duration and adherence introduces uncertainty. Therefore, the assumption of the equivalence of short- and long-term exposure should be made with caution. In addition, the following were not recorded: the burden of white matter disease, the size of the infarct, the NIHSS scores for recurrent IS and sICH, and medications taken at hospital discharge and 90-day follow-up. Only the active compound was recorded for the drug intake. This means that information on dosage, duration, and adherence of antithrombotic and anticoagulant therapy before stroke onset, treatment after initial stroke such as oral anticoagulants and APT not available. Therefore, the effect of secondary prevention with oral anticoagulants on the risk of sICH after the index event could not be analysed. However, considering the very early occurrence of sICH, no effect of oral anticoagulants is assumed.

The inclusion of all symptomatic ICH up to 90 days probably mixed two different entities: haemorrhagic transformation of acute infarct tissue, probably related to revascularisation treatment during hospitalisation, and intraparenchymal haemorrhage, some also outside the infarcted brain tissue, during the 90-day follow-up.

Another limitation is that the DAPT group was smaller than the other groups. Therefore, the power to detect differences in that group might be limited. It is important to acknowledge the limitation that the causal interpretation relies on several key assumptions. Specifically, the validity of the causal model specification and positivity assumption must be ensured. The latter assumption stipulates that there are sufficient numbers of SAPT, nAPT, and DAPT patients in all combinations of covariates. Further assumptions are necessary to ensure the validity of the mediation analyses. Although the temporal sequence—exposure (SAPT, nAPT, DAPT prior to the qualifying event), mediator (acute treatment), and clinical outcome at 90 days—is clearly established, a causal interpretation of the results also requires proper control of confounding factors. Directed acyclic graphs (DAGs) were used to identify potential confounders, and all associations of interest (exposure–outcome, mediator–outcome, and exposure–mediator) were adjusted for relevant covariates. However, the validity of the findings ultimately depends on the completeness and adequacy of the selected covariates.

## Conclusion

Our findings suggest that, regardless of revascularisation treatment, patients with SAPT may have an increased risk of sICH compared to those with nAPT. Patients with DAPT may have an increased risk of recurrent IS and a worse functional outcome than those with SAPT. The majority of sICH occurred within the first few days after admission. Further patients on DAPT are less often given revascularisation treatments.

## Supplementary Material

ds-eso-23969873251369755

## ORCID iDs

Lukas Nussbaum   https://orcid.org/0000-0002-8147-7286

Leo Bonati   https://orcid.org/0000-0003-1163-8133

David J Seiffge   https://orcid.org/0000-0003-3890-3849

Stefan Engelter   https://orcid.org/0000-0003-3855-6234

Alexandros A Polymeris   https://orcid.org/0000-0002-9475-2208

Timo Kahles   https://orcid.org/0000-0002-1569-6376

Alexander Salerno   https://orcid.org/0000-0001-8494-5527

Susanne Wegener   https://orcid.org/0000-0003-4369-7023

Carlo Cereda   https://orcid.org/0000-0002-6479-1476

Manuel Bolognese   https://orcid.org/0000-0003-4757-2834

Friedrich Medlin   https://orcid.org/0000-0002-8477-899X

Nils Peters   https://orcid.org/0000-0001-8451-7389

Maria Ligon   https://orcid.org/0009-0006-8073-4099

Alexander Tarnutzer   https://orcid.org/0000-0002-6984-6958

Gian Marco De Marchis   https://orcid.org/0000-0002-0342-9780

## Data availability statement

The data for this study are from the Swiss Stroke Registry. According to the regulations of the Swiss Stroke Registry Steering Committee, the data is available only on reasoned request. For more information see: https://www.neurovasc.ch/portrait/komitees/swiss-stroke-registry
