# Supplementary material for: Risk of intracranial haemorrhage in patients with acute ischaemic stroke and prior antiplatelet therapy
Source: Eur Stroke J. 2026 Jan 1;11(1):23969873251369755. doi: 10.1093/esj/23969873251369755 (PMC12866221; doi:10.1093/esj/23969873251369755)

**Risk of intracranial haemorrhage in patients with acute ischemic stroke and prior antiplatelet therapy**

**Supplementary Statistical analysis**

Main analysis

The following analyses were performed.

(i) unadjusted: Initially, the effect of APT on each outcome was evaluated using unadjusted regression analyses to calculate the crude odds ratio (OR) and 95% confidence intervals. The following two comparisons were performed: nAPT versus SAPT and DAPT versus SAPT. To maintain consistency with the matched models, these two comparisons were estimated in two separate models instead of fitting one model with APT as a 3-level factor.

(ii) Regression analysis with covariate adjustment: The analysis described above was repeated, including the following variables as covariates: age, sex, mRS before the event, NIHSS at admission, prior TIA or amaurosis fugax, prior AIS or retinal infarction, prior sICH, hypertension, diabetes, hyperlipidaemia, smoking, atrial fibrillation/flutter, coronary heart disease, and a documented left ventricular ejection fraction <35%.

(iii) Propensity score matched analysis: The analysis was repeated as propensity score matched analysis. Matching was performed using the R package “MatchIt”. More precisely, for each comparison of interest (“nAP vs SAPT” and “DAPT vs SAPT”) and each imputed dataset, a 1:1 matched subset was constructed using 1:1 nearest neighbour matching without replacement. The propensity score was estimated using a logistic regression model (generalised linear model with binary error distribution and logit link). The same characteristics as those described above were used for matching. The balance of the matched sets was quantified using the standardised mean difference (SMD). Prior to the analyses described herein, an attempt was made to match across all three groups of interest (nAPT, SAPT, and DAPT). However, this approach was discontinued because of the inadequate balance between the groups.

The same model as described for the adjusted models was fitted to the matched dataset. The results were pooled and presented as described for the other analyses.

To mitigate the risk of bias, a directed acyclic graph (DAG) was created using the web tool from DAGitty.net. The aim was to clarify the possible confounders used to represent prior assumptions about the relationships between variables in a causal structure. The R package “dagitty” was used to assure the absence of cycles. Based on this graph, we used the R function dagitty::adjustmentSets to identify possible sets of adjustment variables which should allow us to estimate the causal association between prior ATP and the occurrence of sICH, given that the causal graph is correct. However, we decided not to limit the covariables to the minimal set according to the DAG. We also included known risk factors for cardiovascular events, even if their role as a cause of exposure was debatable or unlikely.

Sensitivity analysis

The following imputations were performed in these analyses for missing time to event:

1. Patients who died in the hospital but no date was available: 14 patients, median time of all patients who died in the hospital.
2. Patients who suffered a re-stroke in hospital, but no date was available: 3 patients, median time of all patients who suffered a re-stroke in hospital.
3. Patients who suffered a recurrent ischemic stroke after hospitalisation but no date was available: 14 patients, median time of all patients who suffered a re-stroke after hospitalisation.
4. Patients who suffered an sICH after hospitalisation, but no date was available: 23 patients, median time of all patients who suffered an sICH after hospitalisation.

In addition, for patients who suffered the event at baseline day, 0.5 days was imputed as the time to event. Patients without an event and no date of 90-days follow-up were censored at 90 days.

Analysis of possible mediation effect of revascularization treatments

To further assess whether the estimated effect of prior ATP was biased by acute treatment after the qualifying event, the following analyses were performed as described by Baron and Kenny (1986).

1. association between APT and revascularisation treatment,
2. association between revascularisation treatment and outcomes with APT as an additional covariate,
3. including revascularisation treatment as an additional covariate, and
4. discussion of the role of revascularisation treatment as a mediator.

All analyses were performed done using the same models as described for the main analysis, adjusting for patient demographics and pre-existing conditions, as described above.

Handling of missing values

The number of missing values was summarised for each variable. All models were fitted to 50 multiple imputed datasets and pooled using Rubin’s rule. However, if patients died within the first 3 months, we did not impute endpoints which could not be assessed (sICH and recurrent ischemic stroke). Imputation was performed using Chained Equations implemented in the R package mice.

**Supplementary Tables**

Table S1: Swiss Stroke Registry Centers and contributing investigators

The collaborators participated in the SSR and contributed in a significant and documentable manner but did not qualify as regular co-authors. They have confirmed that their names can be listed in any manuscript (i.e. appendix, acknowledgement) arising from this registry.

| **Type** | **Name** | **Investigators** |
| --- | --- | --- |
| Stroke Centre | Inselspital Bern, University Hospital, Department of Neurology |  |
| Stroke Centre | University Hospital Basel, Department of Neurology and Stroke Centre |  |
| Stroke Centre | Cantonal Hospital Aarau, Department of Neurology and Stroke Center | Sandra Clarke  Nicole Kunz |
| Stroke Centre | Cantonal Hospital St. Gallen, Department of Neurology and Stroke Centre |  |
| Stroke Centre | Centre Hospitalier Universitaire Vaudois and University of Lausanne, Department of Neurology | Prof. Patrik Michel |
| Stroke Centre | Hôpitaux universitaires de Geneve, Department of Neurology | PD Dr. Emmanuel Carrera |
| Stroke Centre | University Hospital Zürich, Department of Neurology |  |
| Stroke Centre | Ospedale Regionale Lugano, Stroke Center |  |
| Stroke Centre | Cantonal Hospital Lucerne, Stroke Center |  |
| Stroke Centre | Klinik Hirslanden, Zürich, Stroke Center | Berthold Abel  Klemens Winder  Michael Lehmann  Nikolina Brcina  Janine Schär |
| Stroke Unit | Cantonal Hospital Winterthur | Dr. med. Biljana Rodic |
| Stroke Unit | HFR Cantonal Hospital Fribourg, Stroke Unit and Division of Neurology | Ettore Accolla  David Cuendet  Léonore Jaques  Thierry Badoux |
| Stroke Unit | Cantonal Hospital Muensterlingen, Neurology | Dr. med. Ludwig Schelosky  Dr. Till Betz |
| Stroke Unit | Cantonal hospital Graubuenden Chur, Department of Neurology |  |
| Stroke Unit | Hospital Grabs, Stroke Unit | Florian Lindheimer |
| Stroke Unit | City Hospital Triemli Zuerich, Neurology | Dr. med. Marie-Luise Mono |
| Stroke Unit | Bürgerspital Solothurn, Neurology | Dr. med. Michael Schärer  Dr. med. Robert Bühler |
| Stroke Unit | Neuchatel Hospital Network, Stroke Unit and Division of Neurology |  |
| Stroke Unit | Spitalzentrum Biel, Department of Neurology | Dr. med. Stephan Salmen |
| Stroke Unit | Hôpital Nyon, Stroke Unit | Loraine Fisch  Guillermo Toledo  Angeliki Terpina |
| Stroke Unit | Neurology, Cantonal Hospital of Baden, Baden, Switzerland |  |
| Stroke Unit | Spital Limmattal, Neurology | Dr. med. Guido Schwegler |

Table S2: Baseline table with excluded patients

|  | **Valid for analysis** | **Excluded** | **SMD** | **Missing %** |
| --- | --- | --- | --- | --- |
| Number | 41’113 | 19’577 |  |  |
| age (median [IQR]) | 74 [63.2,82.3] | 77.5 [68.0, 84.3] | 0.238 | 9.4 |
| Sex  - male (%)  - female (%) | 23’826 (58.0)  17’287 (42.1) | 11’031 (56.3)  8’546 (43.8) | 0.034 | 0.2 |
| NIHSS at admission (median [IQR]) | 3.0 [1.0, 8.0] | 4.0 [1.0, 9.0] | 0.048 | 2.1 |
| mRS before event (median [IQR]) | 0.0 [0.0, 1.0] | 0.0 [0.0, 2.0] | 0.228 | 12.3 |
| Past cerebrovascular events  - TIA or amaurosis fugax (%)  - AIS or retinal infarction (%)  - sICH (%) | 1’954 (5.4)  6’483 (15.9)  1’175 (1.8) | 16’078 (92.3)  4’380 (25.1)  2’508 (2.1) | 0.094  0.229  0.021 | 4.3  4.3  4.3 |
| Cerebrovascular risk factors  - Hypertension (%)  - Diabetes (%)  - Hyperlipidaemia (%)  - Smoking (%)  - Atrial fibrillation/flutter* (%)  - Coronary heart disease (%)  - Low ejection fraction (%) | 29’330 (72.1)  8’988 (21.1)  27’510 (66.5)  9’571 (22.1)  6’531 (16)  7’008 (16.1)  13’030 (2.3) | 13’707 (78.5)  6’200 (23.4)  13’549 (65.4)  5’168 (17.1)  7’449 (42.7)  6’034 (22.3)  7’481 (4.2) | 0.150  0.056  0.024  0.126  0.612  0.158  0.108 | 4.2  4.2  4.3  4.6  4.1  4.3  31.9 |
| Treatment  - IVT  - IAT | 11’409 (27.9)  6’477 (15.8) | 2’715 (14.9)  2’766 (15.2) | 0.321  0.018 | 2.6  2.6 |

* The high number of patients with atrial fibrillation/flutter in the excluded patient group was due to anticoagulation therapy, which led to exclusion.

Table S3: Number of patients per year receiving nAPT, SAPT, or DAPT, further stratified by treatment with IVT or EVT.

| **year** | **Total n** | **nAPT n (%)** | **nAPT IVT n (%)** | **nAPT IAT n (%)** | **SAPT n (%)** | **SAPT IVT n (%)** | **SAPT IAT n (%)** | **DAPT n (%)** | **DAPT IVT n (%)** | **DAPT IAT n (%)** |
| --- | --- | --- | --- | --- | --- | --- | --- | --- | --- | --- |
| **2014** | 749 | 439 (58.6%) | 104 (23.7%) | 46 (10.5%) | 289 (38.6%) | 51 (17.6%) | 20 (6.9%) | 21 (2.8%) | 4 (19.0%) | 1 (4.8%) |
| **2015** | 2520 | 1463 (58.1%) | 433 (29.6%) | 202 (13.8%) | 968 (38.4%) | 263 (27.2%) | 135 (13.9%) | 89 (3.5%) | 17 (19.1%) | 14 (15.7%) |
| **2016** | 2560 | 1495 (58.4%) | 463 (31.0%) | 275 (18.4%) | 979 (38.2%) | 309 (31.6%) | 151 (15.4%) | 86 (3.4%) | 20 (23.3%) | 14 (16.3%) |
| **2017** | 4050 | 2421 (59.8%) | 697 (28.8%) | 425 (17.6%) | 1505 (37.2%) | 428 (28.4%) | 235 (15.6%) | 124 (3.1%) | 29 (23.4%) | 21 (16.9%) |
| **2018** | 5135 | 3139 (61.1%) | 893 (28.4%) | 549 (17.5%) | 1821 (35.5%) | 491 (27.0%) | 281 (15.4%) | 175 (3.4%) | 28 (16.0%) | 24 (13.7%) |
| **2019** | 5769 | 3545 (61.4%) | 1019 (28.7%) | 631 (17.8%) | 2036 (35.3%) | 554 (27.2%) | 300 (14.7%) | 188 (3.3%) | 33 (17.6%) | 23 (12.2%) |
| **2020** | 6631 | 4054 (61.1%) | 1090 (26.9%) | 664 (16.4%) | 2367 (35.7%) | 601 (25.4%) | 318 (13.4%) | 210 (3.2%) | 39 (18.6%) | 27 (12.9%) |
| **2021** | 7177 | 4494 (62.6%) | 1269 (28.2%) | 734 (16.3%) | 2454 (34.2%) | 670 (27.3%) | 343 (14.0%) | 229 (3.2%) | 41 (17.9%) | 17 (7.4%) |
| **2022** | 6293 | 4009 (63.7%) | 1211 (30.2%) | 691 (17.2%) | 2065 (32.8%) | 614 (29.7%) | 312 (15.1%) | 219 (3.5%) | 38 (17.4%) | 24 (11.0%) |

Table S4: Time-to-event analysis of primary outcome sICH. Values are OR (boxes in red OR <1, boxes in green OR >1), confidence intervals and p (bold p<0.01).

|  | without consideration of revascularisation treatment | | adjusted for IVT | | adjusted for EVT | | |
| --- | --- | --- | --- | --- | --- | --- | --- |
|  | unadjusted | covariate adjusted | unadjusted | covariate adjusted | unadjusted | covariate adjusted | |
| nAPT vs SAPT | **0.78 (0.66 – 0.91) <0.01** | **0.75 (0.62 – 0.90) <0.01** | **0.75 (0.64 – 0.88) <0.01** | **0.75 (0.63 – 0.90) <0.01** | **0.72 (0.62 – 0.85) <0.01** | **0.74 (0.62 – 0.89) <0.01** | |
| DAPT vs SAPT | | 0.83 (0.53 – 1.30) 0.41 | 0.88 (0.56 – 1.40) 0.59 | 1.00 (0.64 – 1.58) 1.00 | 1.02 (0.64 – 1.62) 0.94 | 0.88 (0.56 – 1.38) 0.56 | 0.93 (0.59 – 1.47) 0.76 |

Table S5: Time-to-event analysis of secondary outcomes. Values are OR (boxes in red OR <1, boxes in green OR >1), confidence intervals and p (bold p<0.01).

|  | | without consideration of revascularisation treatment | | adjusted for IVT | | adjusted for EVT | | |
| --- | --- | --- | --- | --- | --- | --- | --- | --- |
|  |  | unadjusted | covariate adjusted | unadjusted | covariate adjusted | unadjusted | covariate adjusted | |
| Recurrent IS | nAPT vs SAPT | **0.74 (0.67 – 0.82) <0.01** | 0.92 (0.81 – 1.03) 0.15 | **0.74 (0.67 – 0.82) <0.01** | 0.92 (0.81 – 1.03) 0.15 | **0.73 (0.67 – 0.81) <0.01** | 0.91 (0.81 – 1.03) 0.14 | |
|  | DAPT vs SAPT | **1.50 (1.23– 1.83) <0.01** | **1.38 (1.12 – 1.69) <0.01** | **1.49 (1.22 – 1.83) <0.01** | **1.38 (1.12 – 1.69) <0.01** | **1.50 (1.23 – 1.84) <0.01** | **1.38 (1.13 – 1.70) <0.01** | |
| All-cause mortality | | nAPT vs SAPT | **0.70 (0.66 – 0.75) <0.01** | 1.00 (0.93 – 1.08) 0.95 | **0.70 (0.65 – 0.74) <0.01** | 1.00 (0.92 – 1.08) 0.97 | **0.68 (0.63 – 0.72) <0.01** | 1.00 (0.93 – 1.08) 0.96 |
|  | | DAPT vs SAPT | 0.88 (0.73 – 1.06) 0.19 | 0.95 (0.78 – 1.17) 0.63 | 0.92 (0.76 – 1.11) 0.38 | 0.94 (0.77 – 1.15) 0.54 | 0.91 (0.76 – 1.10) 0.34 | 0.96 (0.78 – 1.17) 0.67 |

**Supplementary Figures**

Figure S1: Proportion of patients with nAPT, SAPT and DAPT per year (blue = nAPT, purple = SAPT, red = DAPT)


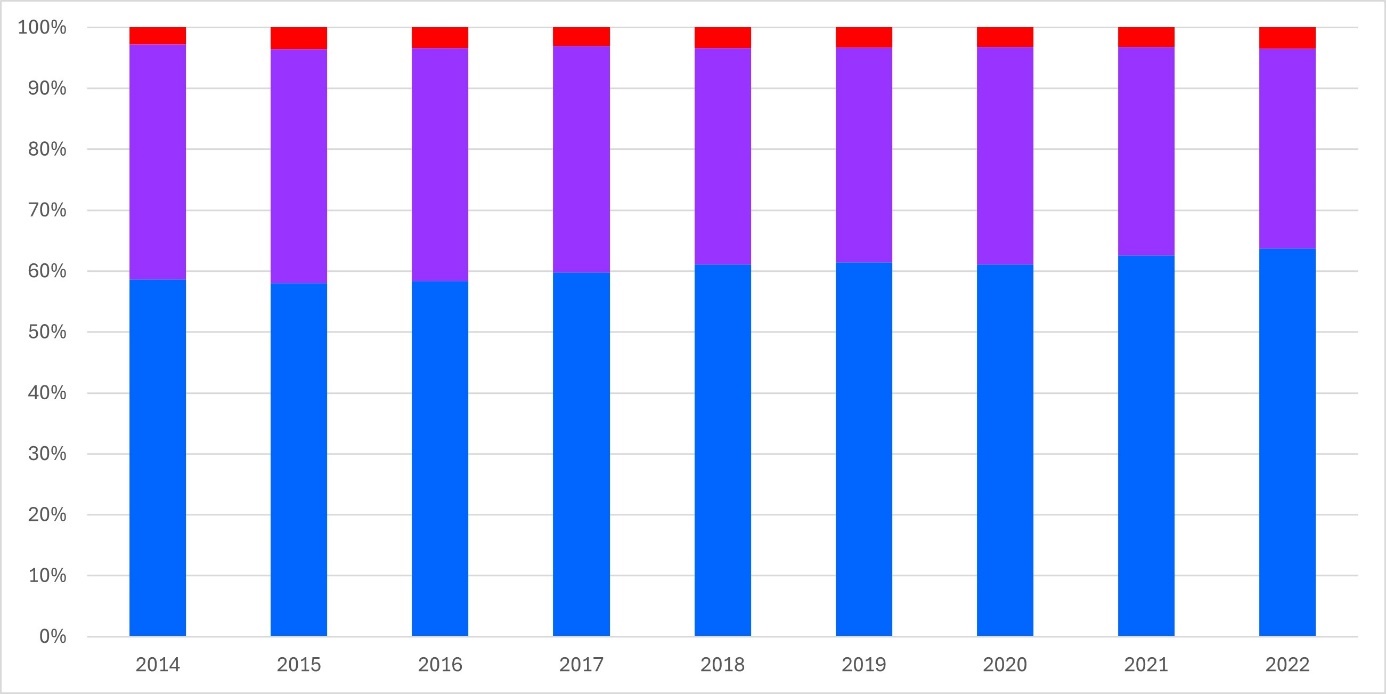


Figure S2: Probability of receiving IVT or EVT depending on prior APT. Group comparison of nAPT versus SAPT and DAPT versus SAPT (bars = 95% Confidence Interval).


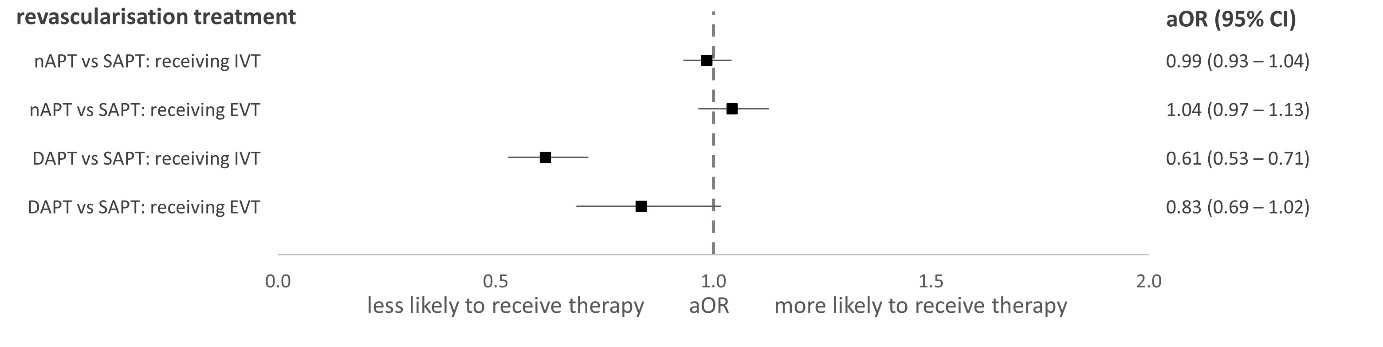


Figure S3: Proportion of patients with IVT by year (blue = nAPT, purple = SAPT, red = DAPT)


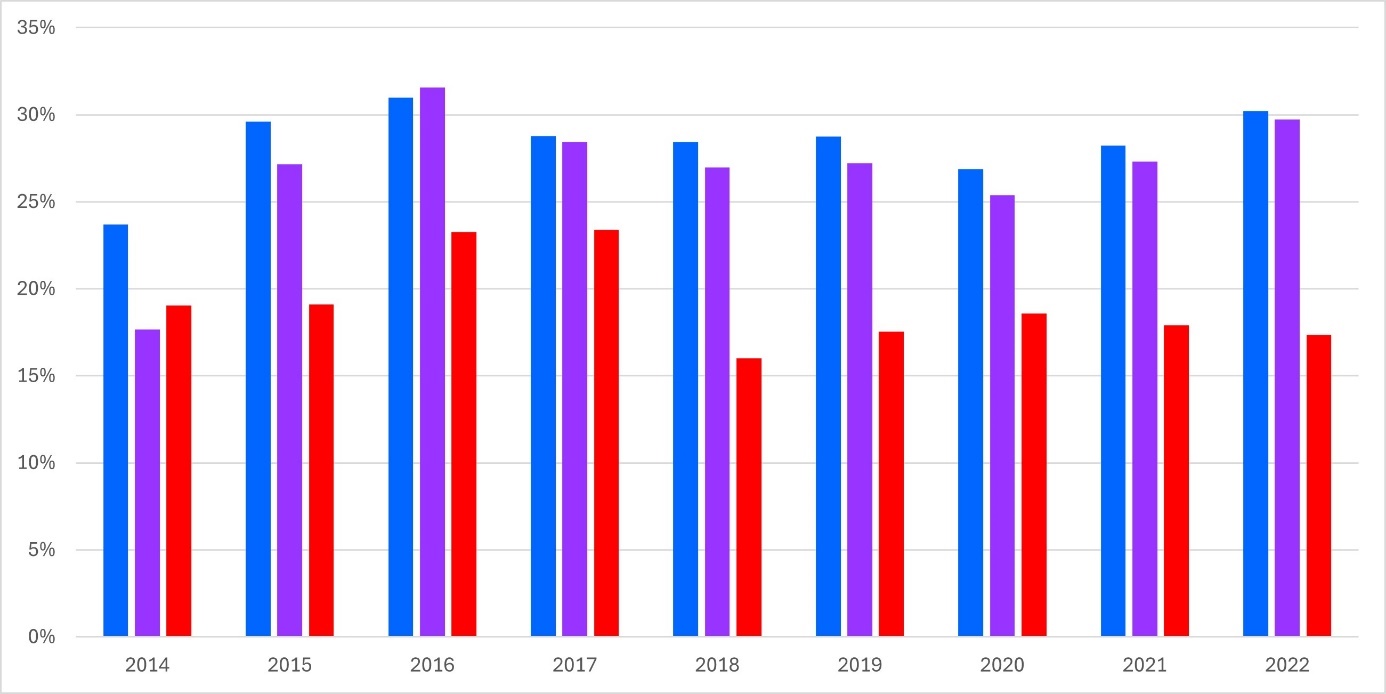


Figure S4: Proportion of patients with IAT by year (blue = nAPT, purple = SAPT, red = DAPT)


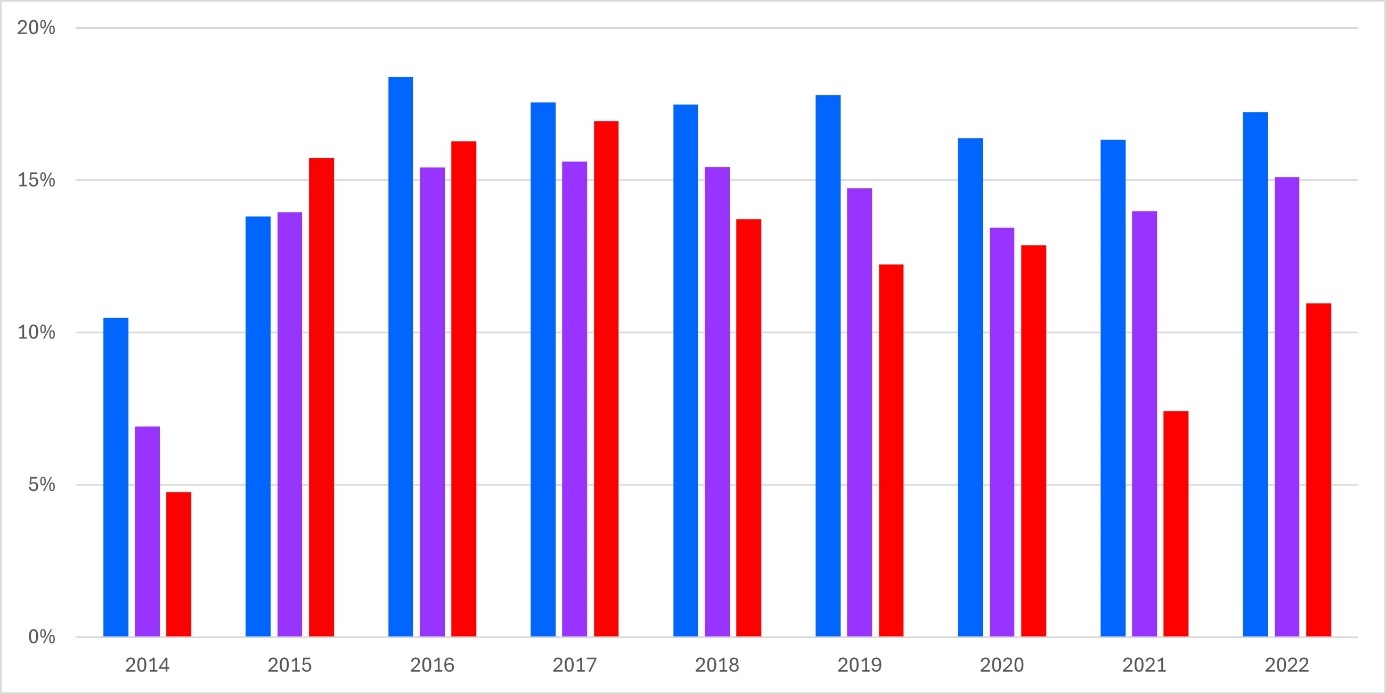


Figure S5: Directed acyclic graph for the primary endpoint sICH. Circles in green = exposure, in blue = outcome, in grey = not observed or not adjusted, in white = adjusted, green arrow = causal path). Treat OAC/APT was summarised because all patients after an AIS will generally receive APT, except for those who have an indication for OAC.


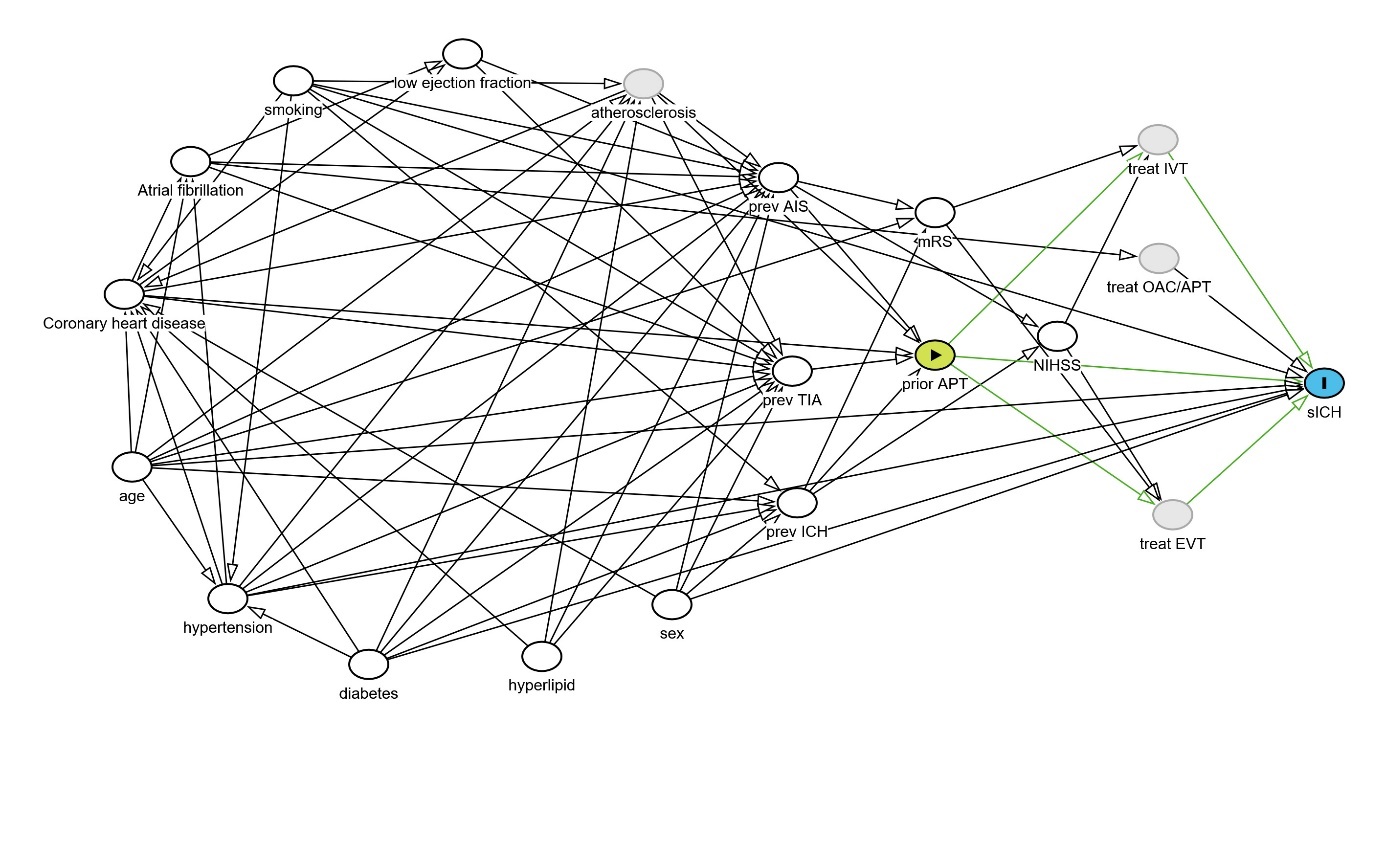


Figure S6: Odds Ratios of primary outcome sICH (bars = 95% Confidence Interval).


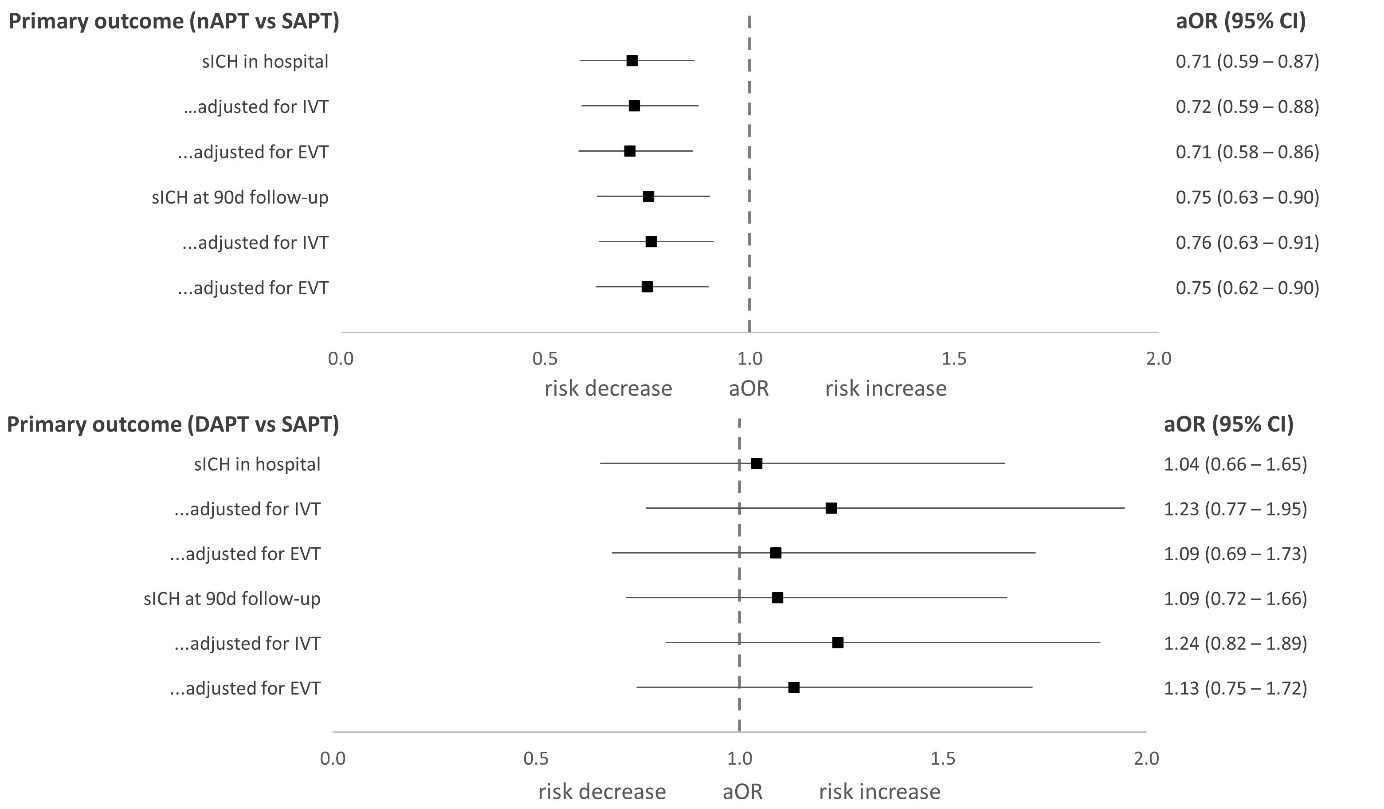


Figure S7: Odds Ratios of secondary outcomes (bars = 95% Confidence Interval).


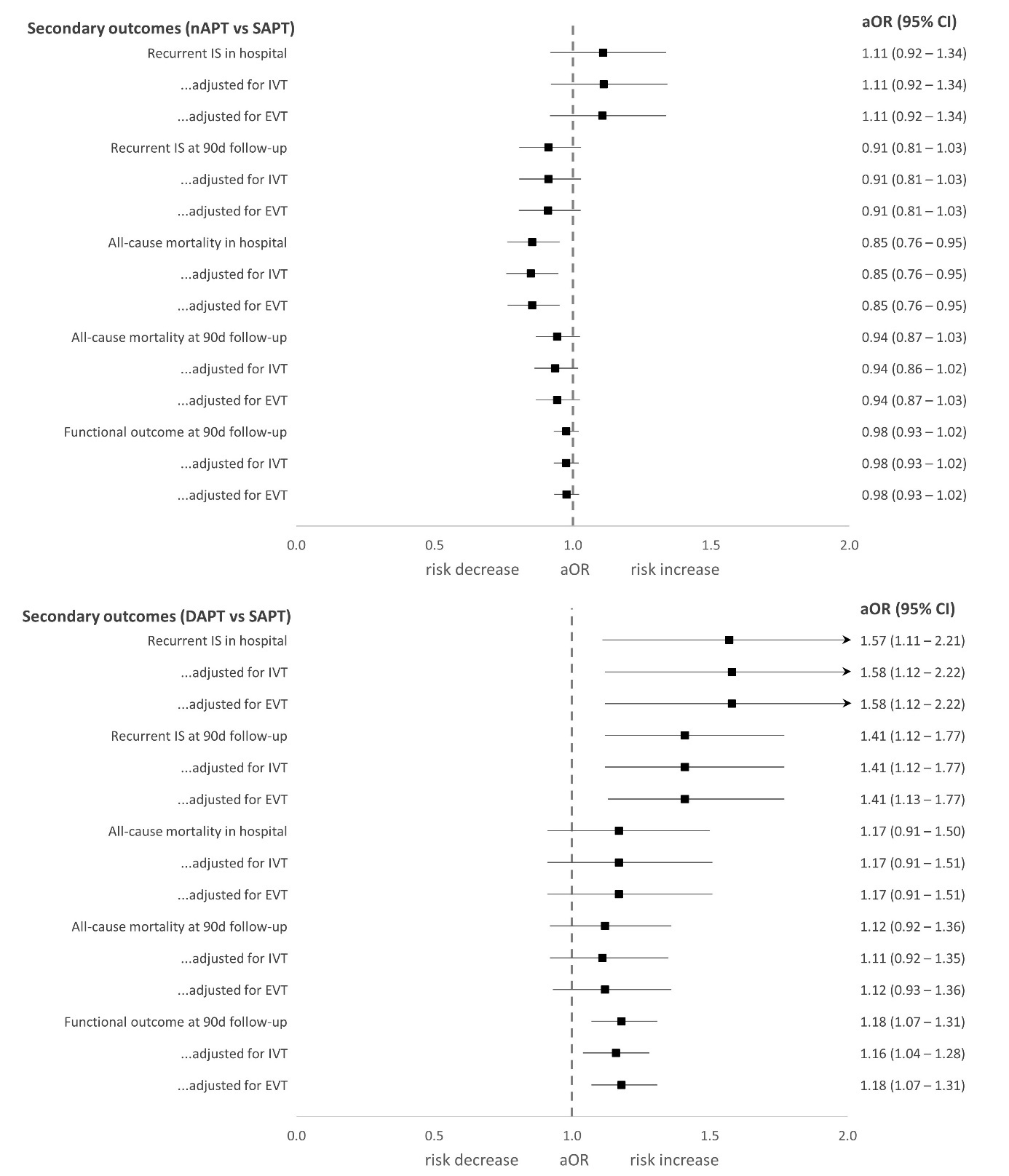

Supplement: ds-eso-23969873251369755 [file ds-eso-23969873251369755.zip › sj-docx-1-eso-10.1177_23969873251369755.docx]
